# The recognition of gaming disorder in China: a case series of 223 patients

**DOI:** 10.7717/peerj.10827

**Published:** 2021-02-04

**Authors:** Tianli Shao, Xinxin Chen, Shucai Huang, Zhenjiang Liao, Shuhong Lin, Jing Qi, Yi Cai, Qiuping Huang, Hongxian Shen

**Affiliations:** 1 National Clinical Research Center for Mental Disorders, and Department of Psychiatry, the Second Xiangya Hospital of Central South University, Changsha, Hunan, China; 2 Institute of Mental Health of Central South University, Chinese National Technology Institute on Mental Disorders, Hunan Key Laboratory of Psychiatry and Mental Health, Hunan Medical Center for Mental Health, Changsha, Hunan, China; 3 Department of Psychiatry, Comorbid Somatic Diseases, Kangning Hospital of Shenzhen, Shenzhen, Guangdong, China; 4 Department of Psychiatry, the Fourth People’s Hospital of Wuhu, Wuhu, Anhui, China; 5 Department of Psychiatry, Brain Hospital of Hunan Province, Changsha, Hunan, China

**Keywords:** Gaming disorder, Diagnosis, Recognition, Patients

## Abstract

**Background:**

Internet gaming disorder (IGD) was listed in the appendix of the Diagnostic and Statistical Manual of Mental Disorders, Fifth Edition (DSM-5) as a condition requiring further study in 2013, and gaming disorder (GD) was considered a mental disorder and listed in the 11th International Classification of Diseases Manual (ICD-11) in 2018. The study aims to obtain preliminary knowledge of the recognition of GD in China.

**Methods:**

A total of 223 Chinese patients who met both the ICD-11 and DSM-5 criteria for GD participated in the study, and a self-administered questionnaire was used to collect demographic information, gaming use characteristics, and previous diagnoses.

**Results:**

The average age of patients with GD was 20.5 years, and 71.3% were male. Most patients were diagnosed with emotion-related disorders at their first psychiatric visit: mood disorders (59.2%), bipolar affective disorder (18.4%), depressive episode (12.6%), and anxiety disorder (4.9%). Among the adolescent patients with a first diagnosis of mood disorders, 71.2% and 33.3% were diagnosed with bipolar affective disorder and personality disorders, respectively, at subsequent visits. Overall, after the first visit, the most common diagnosis was bipolar affective disorder (61.0%), followed by personality disorders (34.1%), mood disorders (17.0%), depressive episode (17.0%), and other disorders. Only three patients had Internet overuse.

**Conclusion:**

The identification rate of GD is extremely low in routine psychiatric clinical practice in China. Most patients with GD were previously misdiagnosed with emotion-related disorders. Psychiatrists should be trained to improve their ability to recognize and manage GD.

## Introduction

Released by the China Internet Network Information Center (CNNIC), the 44th China Statistical Report on Internet Development showed that the number of Internet users in China was 854.49 million, of whom 493.56 million (57.8%) were online game users and 467.56 million (55.2%) were mobile online game users until June 2019 (Chinese Internet Network Information Center (CNNIC), 2019). A recent study reported that 43.7% of undergraduate students had at least one type of Internet-related addiction, such as online gaming addiction, online social networking addiction, and Internet addiction (Tang, Koh & Gan, 2017). Studies have shown that the prevalence of Internet gaming addiction among adolescents all around the world ranged from approximately 0.7–27.5% (Chung, Sum & Chan, 2019; King et al., 2020; Lecardeur, 2013; Li et al., 2018; Mak et al., 2014; Mann et al., 2016; Mihara & Higuchi, 2017; Müller et al., 2015; Rehbein et al., 2015; Xiang et al., 2020; Yu & Cho, 2016). Previous research has also revealed that the clinical features and neural mechanisms of gaming disorder (GD) are similar to those of drug addiction (Kuss, Pontes & Griffiths, 2018; Weinstein, Livny & Weizman, 2017). GD negatively affects people’s work, study, and social and family life, leading to a series of physiological, psychological, and social problems (Gentile et al., 2011; Lecardeur, 2013; Mamun et al., 2020; Schneider, King & Delfabbro, 2017; Tang, Koh & Gan, 2017). With the rapid development of online games, GD has become an increasingly recognized behavioral addiction, attracting much attention because of its serious impact on health.

In 2013, Internet gaming disorder (IGD) emerged as a new mental disorder in the third part of the Diagnostic and Statistical Manual of Mental Disorders, Fifth Edition (DSM-5) of the American Psychiatric Association, which requires further research (Petry et al., 2014). The 11th International Classification of Diseases Manual (ICD-11) issued by the World Health Organization (WHO) in 2018 added GD as a diagnostic category in the chapter on mental, behavioral, and neurodevelopmental disorders (WHO, 2018). The diagnostic criteria for GD both in the DSM-5 and ICD-11 considered the loss of control over game use as a basic symptom. However, the ICD-11 further emphasizes serious symptoms of GD (Jo et al., 2019). IGD and GD were included in the DSM-5 and ICD-11 diagnostic systems, respectively, which is helpful for clinical diagnosis by psychiatrists and the awareness by the public of this new mental disorder.

There have been many studies on “Internet gaming disorder”; however, most of them used screening scales rather than strict clinical diagnoses. Few studies have been conducted based on clinical diagnoses using the standardized criteria of the two diagnostic systems (Przybylski, Weinstein & Murayama, 2017). GD is not commonly diagnosed by clinical psychiatrists in China. In China, clinical doctors usually make their diagnoses on the basis of the ICD-10, the former version that does not contain GD, resulting in difficulties in the identification of patients with GD. This study investigated the current status of the recognition of Chinese patients with GD and hopefully provides some reference information for the ICD-11 diagnostic criteria, which is scheduled to be effective from January 1, 2022.

## Materials and Methods

### Participants

As the National Clinical Research Center for Mental Disorders, the Second Xiangya Hospital of Central South University is the only institution of the WHO cooperative center for addictive behaviors in China, providing outpatient services to patients in various provinces of China with a variety of addictive disorders. The current study recruited patients from the medical addiction outpatient department of the Second Xiangya Hospital from February 2018 to July 2019.

The Institutional Review Board of the Second Xiangya Hospital granted ethical approval to carry out the study within its facilities (2017; No. 048). Written consent was obtained from all participants and their parents (when necessary).

### Measures

All diagnoses were made by a senior and experienced psychiatrist who was a professor in clinical addiction medicine. Patients had a history of playing games and met the GD diagnostic criteria of both the DSM-5 and the ICD-11, which was stricter (Jo et al., 2019). Patients who persistently and recurrently used the Internet to engage in games, often played games with other players, and were clinically impaired or distressed by five or more of the nine symptoms in the past 12 months were considered to meet the DSM-5 diagnostic criteria (Petry et al., 2014). According to the ICD-11, patients with GD show three symptoms: prioritizing gaming over other activities, having impaired control over gaming, and continuing to game or escalating gaming behavior despite negative consequences (WHO, 2018). We excluded patients with the following characteristics: a history or current episode of other primary psychiatric diseases, other substance abuse, and a history of head trauma or other neurological diseases.

A self-administered questionnaire was used to collect demographic information (age, gender, education), total duration of online gaming (years), duration of online gaming per day (hours), types of games (League of Legends, Honor of Kings, Player Unknown’s Battlegrounds, and others), current life status (in school, school suspension, at home, others), and previous diagnoses (diagnosis at the first psychiatric visit and subsequent diagnoses).

### Statistical analysis

Demographic data, game-related characteristics, and previous diagnoses were described. Continuous variables were presented as mean values with standard deviations, and categorical variables were presented as frequencies and percentages. SPSS software version 20.0 was used for all analyses.

## Results

The average age of the 223 patients was 20.5 years (standard deviation (SD):5.1, range: 13–33 years), and 71.3% were male. The mean number of years spent playing games was 5.7 years (SD:2.6), and the mean number of daily gaming hours was 13.1 h (SD:1.7). All patients played Honor of Kings, 96.9% played Player Unknown’s Battlegrounds, and 73.5% played League of Legends. Regarding current status, 42.2% of patients had stopped schooling, 38.1% were on medical leave, and 14.3% were still in school. Detailed sociodemographic and game-related characteristics are shown in Table 1.

**Table 1:** Sociodemographic and gaming playing characteristics.

	Total (*N* = 223)
Age	20.5 ± 5.1
<18 years	95 (42.6%)
≥18 years	128 (57.4%)
Sex	
Male	159 (71.3%)
Female	64 (28.7%)
Education	
Primary school	5 (2.2%)
Middle school	54 (24.2%)
High school	69 (30.9%)
College	95 (42.6%)
Years of playing games	5.7 ± 2.6
Hours of daily gaming	13.1 ± 1.7
Main gaming category	
League of Legends	164 (73.5%)
Honor of Kings	223 (100%)
Player Unknown’s	216 (96.9%)
Current status	
In school	32 (14.3%)
School suspension	94 (42.2%)
At home	85 (38.1%)
Others	12 (5.4%)

As shown in Table 2, the percentages of patients diagnosed with specific disorders at the first visit were as follows: mood disorders, 59.2%; bipolar affective disorder, 18.4%; depressive episode, 12.6%; schizophrenia, 1.3%; anxiety disorder, 4.9%; and unspecified non-organic psychosis, 3.1%. After the first diagnosis of mood disorders (as shown in Table 3), 71.2% were diagnosed with bipolar affective disorder, 33.3% with personality disorders, and 19.7% with unspecified non-organic psychosis.

**Table 2:** Diagnosis at first psychiatric visit.

	Total (*N* = 223)
Mood disorders	132 (59.2%)
Bipolar affective disorder	41 (18.4%)
Depressive episode	28 (12.6%)
Anxiety disorder	11 (4.9%)
Unspecified nonorganic psychosis	7 (3.1%)
Schizophrenia	3 (1.3%)
Other mental problems	1 (0.4%)

**Table 3:** Subsequent diagnoses of patients with the first diagnosis of adolescent emotional disorders.

Disorders	Mood disorders	Personality disorders	Bipolar affective disorder	Schizophrenia	Depressive	Anxiety disorder	Unspecified nonorganic psychosis	Nonorganic sleep disorders	Conduct disorders	Schizotypal disorder	Internet overuse
Total (*N* = 132)	7 (5.3%)	44 (33.3%)	94 (71.2%)	7 (5.3%)	14 (10.6%)	8 (6.1%)	26 (19.7%)	7 (5.3%)	6 (4.5%)	5 (3.8%)	2 (1.5%)

In total, as shown in Table 4, 61.0% of patients were diagnosed with bipolar affective disorder between the first visit and the most recent visit, 34.1% had personality disorders, 17.0% had mood disorders or depressive episode, 16.1% had unspecified non-organic psychosis, 8.5% had anxiety disorder, 6.7% had non-organic sleep disorders, and 5.4% had schizophrenia.

**Table 4:** Previous diagnoses (Diagnoses in the past except diagnosis at first visit).

	Total (*N* = 223)
Bipolar affective disorder	136 (61.0%)
Personality disorders	76 (34.1%)
Mood disorders	38 (17.0%)
Depressive episode	38 (17.0%)
Unspecified nonorganic psychosis	36 (16.1%)
Anxiety disorder	19 (8.5%)
Nonorganic sleep disorders	15 (6.7%)
Schizophrenia	12 (5.4%)
Conduct disorders	11 (4.9%)
Schizotypal disorder	10 (4.5%)
Internet overuse	3 (1.3%)

## Discussion

To the best of our knowledge, this is the first study to investigate the current diagnosis of GD in psychiatric clinical practice in China, based on psychiatrists’ encounters with patients. We found that patients with GD were more likely to be young and male. Patients with GD were commonly misdiagnosed with emotion-related disorders at their first psychiatric visit, including mood disorders, bipolar affective disorder, depressive episode, and anxiety disorder. After the first visit, the most common diagnosis was bipolar affective disorder, followed by personality disorders, mood disorders, depressive episode, and other disorders. In contrast, only three patients were diagnosed with Internet overuse.

In this study, patients with GD were predominantly male, which is consistent with the findings of prior studies. Previous studies reported that males were more likely to develop online game addiction than females (Bonnaire & Baptista, 2019; Lee et al., 2018; Mihara & Higuchi, 2017; Paulus et al., 2018; Tang, Koh & Gan, 2017). This gender difference could be explained by feminine empathy. Females feel guilty about violent games and show less enthusiasm for games (Anderson & Murphy, 2003). Previous studies found that females and males were more addicted to online social networking, and online gaming, respectively (Tang, Koh & Gan, 2017). Furthermore, some mental characteristics of males, such as high levels of novelty-seeking and impulsivity, may be associated with higher rates of GD (Bonnaire & Baptista, 2019). According to a functional magnetic resonance imaging study, some neural mechanisms explain that compared with females, males could be more vulnerable to developing GD (Dong et al., 2018). Students accounted for 42.6% of the sample in the survey, with a mean age of 20.5 years. According to the average number of years spent playing games, we can speculate that most of these students had been playing online games since their middle school years, suggesting that teenagers are a group at high risk of GD and should be the target population for prevention programs (Min Zhao, 2018).

In the present study, most patients were diagnosed with mood disorders at their first visit to a psychiatrist. Only three patients (1.3%) were diagnosed with Internet overuse after the first visit, indicating a very low recognition rate of GD in China. There were some possible reasons for this finding. First, the concepts of the pathways leading to GD and the treatments of GD are not entirely clear. GD may be a dangerous disorder involving a complex psychosocial background (Paulus et al., 2018). Second, there are no standard diagnostic criteria for psychiatrists, who may ignore GD diagnosis. Although IGD is listed in the DSM-5, the diagnostic system commonly used in China is the ICD-10, which does not contain the GD diagnostic criteria. Third, there are no effective treatments for GD, and psychiatrists may tend to make a diagnosis other than GD. Fourth, clinicians who ignore the patient’s history of engaging in games may misdiagnose GD. Therefore, it is essential to apply the diagnostic criteria for GD in Chinese addiction clinical practice as soon as possible.

As GD is comorbid with some mental disorders, including anxiety disorder, depressive episode, and irritability, we excluded these participants at the beginning of the study. In this study, patients with GD were often misdiagnosed with mood disorders, bipolar affective disorder, personality disorders, and other mental disorders. These misdiagnoses may result from a series of clinical symptoms of GD. GD has been reported to be associated with somatization, emotional problems (depression, anxiety, phobic anxiety, irritability) (Tang, Koh & Gan, 2017), behavioral problems (hostility, suicidal ideation, suicide), personality characteristics (high impulsivity, neuroticism, paranoid, extraversion, and agreeableness) (Kim et al., 2016; Sung et al., 2013), social function impairment (interpersonal sensitivity, academic failure), obsession-compulsion, and even psychotic disorders (Min Zhao, 2018). Gamers showed withdrawal symptoms with irritability and restlessness after cessation of gaming use (Kaptsis et al., 2016). More patients with GD and their families visit clinicians because of the emotional and behavioral symptoms induced by GD rather than gaming problems. Additionally, in this study, most participants had participated in online games since their teenage years, and mood disorders are diagnoses commonly used in China for adolescents with mood-related problems, such as depressive episode and anxiety disorder, which may protect them, to some extent, from discrimination. Since GD is a mental health disorder with complex clinical presentations, it is necessary for psychiatrists to gain more knowledge about it.

As shown in this study, only 14.3% of patients were in school, and the rest had either dropped out of school or stayed at home due to their mental health conditions. This clearly indicated the social functional impairments resulting from GD. Previous research showed that many teenagers gave up schooling due to GD (Bargeron & Hormes, 2017). Pathological gaming was negatively associated with academic performance and affected self-esteem and self-confidence (Toker & Baturay, 2016). This research suggests that GD leads to negative social consequences and academic failure, which requires clinical and social attention. A misdiagnosis would result in mistreatment and delay in receiving appropriate treatment. Therefore, it is essential to diagnose and treat patients with GD as early as possible.

There were some limitations to this study. First, as the data were collected retrospectively, there might be some recall bias. Second, more details of diagnoses and treatment should be recorded, including the psychiatric symptoms and the effectiveness of current and previous treatments, which are helpful for understanding the management status of Chinese patients with GD. Lastly, more follow-up research should be conducted to determine the prognosis of GD.

## Conclusions

In conclusion, GD is poorly recognized in psychiatric clinical practice in China. Most patients with GD had previously been misdiagnosed with emotion-related disorders. As GD is a complex mental disorder, psychiatrists should be trained to increase their awareness and obtain more knowledge about GD. It is necessary to apply the new diagnostic criteria for GD in clinical work as soon as possible.

## Supplemental Information

10.7717/peerj.10827/supp-1 Supplemental Information 1 A case series of 223 patients.

10.7717/peerj.10827/supp-2 Supplemental Information 2 Research Protocol.

## Additional Information and Declarations

### Data Availability

The following information was supplied regarding data availability: Raw measurements are available in Supplemental Files.
