# Supplementary material for: The recognition of gaming disorder in China: a case series of 223 patients
Source: PeerJ. 2021 Feb 4;9:e10827. doi: 10.7717/peerj.10827 (PMC7868065; doi:10.7717/peerj.10827)
Supplement: Supplemental Information 2 [file peerj-09-10827-s002.pdf]

研究方案

一、目的意义

1、研究意义

近年来,随着网络游戏开发的日新月异,以游戏障碍为代表的网络成瘾已经成为了一种新的行为成瘾现象,因对成瘾者心身健康和社会功能产生的严重影响而受到关注:Gentile 发现,在美国 8-18 岁的青少年中,8.5%的人会因玩游戏而影响正常的社会功能;在韩国和德国等国家调查研究中也发现游戏障碍的发生率在 1.2%-9.2%。中国的情况同样不容乐观, 第 38 次中国互联网络发展状况统计报告显示:截至 2016 年 6 月,中国网民规模达到 71000 万;网络游戏用户规模达到 39108 万,占 55.1%。中国青少年网络协会 2009 年发布的中国青少年网瘾报告显示，我国城市青少年网民中网瘾青少年约占 14.1%，人数约为 2404 万；青少年网络成瘾以网络游戏成瘾者居多,占 47.9%。网络成瘾者的网络滥用和网络依赖显著地影响到他们的工作学习社交和家庭生活,并导致行为异常心理障碍和神经功能紊乱等。而游戏障碍的治疗难度堪比戒毒,需要防-治双管齐下,因此早期诊断和干预尤为重要。

2、国内外发展现状与趋势

目前,网络成瘾中的游戏障碍已成为备受关注的行为成瘾类型之一,其导致的心身健康问题已不容忽视。美国精神病学协会《精神障碍诊断和统计手册》第五版（DSM-5）将游戏障碍（IGD）作为“尚需要进一步研究和观察的精神障碍”列在附录中。以往有很多关于网络游戏障碍的研究，但大多数研究者使用筛查量表，而不是严格的临床诊断。IGD 目前在中国临床精神病医生的诊断中并不常见，且临床医生通常根据 ICD-10（无 IGD 的版本）进行诊断，以上情况导致我国 IGD 患者的识别困难。因此，根据 DSM-5 标准化诊断系统对于 IGD 进行临床诊断的研究十分重要。

二、主要目标及研究内容

1、项目实施的目标

本研究拟通过资深精神科医生严格的临床诊断,探索中国医生 IGD 患者的识别现状,对未来 IGD 诊断标准的推广和发展提供一些参考信息；探索 IGD 的相关因素及可能的不良后果，以提高社会各界对该类问题的关注和重视；发表 SCI 论文 1 篇。

2、研究内容、关键技术和创新点

2.1 研究内容

本研究内容包括：以 DSM-5 网络游戏障碍诊断条目为标准，探究中国门诊 IGD 患者的诊断现状;探究游戏障碍相关因素以及可能导致的消极后果。

2.2 拟采取的研究方法

2.2.1 研究对象

本研究的从中南大学湘雅二医院药物成瘾门诊部招募被试者，预计入组 300 人。具体入组和排除标准如下：

入组标准：

a. 持续和反复使用互联网进行游戏；

b. 在过去 12 个月内出现 DSM-5 诊断标准 9 条诊断标准中的 5 条（或更多）；

c. 能够参加并愿意提供参与研究的知情同意(若被试者年龄小于 18 岁，需要获得监护人的知情同意）。

排除标准：

a.有其他原发性精神疾病的病史或当前发作;

b.其他药物滥用史;

c.头部外伤或其他神经系统疾病病史。

2.2.2 临床评估工具

a. 人口学资料：采集被试者的年龄、性别、文化程度等个人信息。

b. 网络游戏相关特征：采集被试者使用网络游戏的情况，包括网络游戏总持续时间（年）、每天网络游戏持续时间（小时）、游戏类型（传奇联盟、国王荣誉、玩家未知战场等）。

c. 当前生活状况：调查被试者当下是处于在校、停学、在家或其他中的何种状态。

d. 以往的诊断：采集被试者于精神科第一次就诊及后来的就诊中的临床诊断。

e. DSM-5 网络游戏障碍：过去 12 个月，满足 DSM-5 的 9 条诊断标准中的 5 条及以上者为 IGD 诊断阳性。

2.2.3 研究实施

本研究先向伦理委员会提出申请，得到批准后正式开始实施；由一名经验丰富、从事成瘾医学研究的精神科教授对被试者进行评估，按照入组标准和排除标准筛选入组人员，并取得被试的书面知情同意；收集被试者的社会人口学资料，包括年龄、性别、文化程度；游戏使用相关特征：网络游戏总持续时间（年）、每天网络游戏持续时间（小时）、游戏类型（传奇联盟、国王荣誉、玩家未知战场等）；以及当前生活状况，包括在校、停学、在家、其他；和以前的在精神科就诊时的诊断，包括第一次就诊时的诊断和后来的诊断。

2.2.4 统计方法

采用 SPSS17.0 进行数据整理和分析。对被试的一般资料等情况使用描述性统计；采用卡方检验、独立样本 t 检验对不同组间的被试者进行比较。

2.3 创新点

首次在中国临床诊疗过程中对 IGD 的诊断进行研究，用于调查中国精神科医生对 IGD 患者的识别情况，为未来 IGD 诊断标准化的发展提供指导。

2.4 技术路线

- **入组** 依据DSM-5对被试者进行评估
- **社会人口学信息** 年龄、性别、文化程度
- **游戏相关特征** 网络游戏总持续时间、每天网络游戏持续时间、游戏类型
- **既往精神科诊断**
- **学业状况** 在校、休学、在家、其他
